# Patterns, Barriers and Facilitators of Responsiveness to Text Message Medication Reminders Among Youth Living with HIV in Southwest Nigeria

**DOI:** 10.3390/tropicalmed10050137

**Published:** 2025-05-17

**Authors:** Mobolanle Balogun, Aniekan E. Ulor, Mayowa Odofin, Olufunmilola Idowu, Mmeli V. Chukwu, Abiola Aina, Oluwanifemi Adeshina, Hameed Adelabu, Lisa M. Kuhns, Amy K. Johnson, Kehinde M. Kuti, Nadia A. Sam-Agudu, Titilope Badru, Marbella Cervantes, Robert Garofalo, Babafemi Taiwo, Alani S. Akanmu

**Affiliations:** 1 Department of Community Health and Primary Care, College of Medicine, University of Lagos, Lagos 12003, Nigeria; 2 Department of Community Health, Lagos University Teaching Hospital, Lagos 12003, Nigeria; 3 Department of Haematology and Blood Transfusion, Lagos University Teaching Hospital, Lagos 12003, Nigeria; 4 Department of Pediatrics, Northwestern University Feinberg School of Medicine, Chicago, IL 60611, USA; 5 Division of Adolescent and Young Adult Medicine, Ann and Robert H Lurie Children’s Hospital of Chicago, Chicago, IL 60208, USA; 6 Infectious Disease Institute, College of Medicine, University of Ibadan and Staff Medical Services Department, University College Hospital, Ibadan 200285, Nigeria; 7 International Research Centre of Excellence, Institute of Human Virology Nigeria, Abuja 900231, Nigeria; 8 Department of Paediatrics and Child Health, University of Cape Coast School of Medical Sciences, Cape Coast 00233, Ghana; 9 Global Pediatrics Program and Division of Infectious Diseases, Department of Pediatrics, University of Minnesota Medical School, Minneapolis, MN 55455, USA; 10 Division of Infectious Diseases and Institute for Global Health, Northwestern University, Chicago, IL 60611, USA; 11 Department of Haematology and Blood Transfusion, College of Medicine, University of Lagos, Lagos 12003, Nigeria

**Keywords:** adolescent, medication adherence, HIV, text messaging, Nigeria

## Abstract

The iCARE Nigeria study is evaluating a daily text message medication reminder intervention (TXTXT) to improve viral suppression and medication adherence among youth living with HIV (YLH), aged 15–24 years. In this sub-study, we evaluated text message responsiveness (text-back) at 24 weeks of the intervention as an indicator of engagement, as well as barriers and facilitators at one of six clinical study sites. Differences in responses by age group, birth sex, schooling status, education, mode of infection, and weekend/weekday and holiday/non-holiday periods were analyzed using *t*-test and multiple linear regression. Focus group discussions were conducted among three groups (low, average, and high text message responsiveness) and analyzed using a rapid content analysis approach. Overall, TXTXT responsiveness was 26.5% (4606/17,367); older age (18–24 years) and weekdays (versus weekends) were significantly associated with higher responsiveness. Facilitators included being comfortable receiving personalized text messages. Barriers included a lack of airtime and messages received late. Overall, text-back responsiveness to daily medication adherence messages among YLH was low, better among older participants, and higher on weekdays. Addressing barriers and promoting facilitators may improve responsiveness.

## 1. Introduction

Globally, in 2023, an estimated 360,000 youths aged 15 to 24 years were newly infected with HIV [1], and 14,000 adolescents aged 10–19 years died from HIV [2]. More than 80% of youths living with HIV (YLH) live in high-burden African countries [3], and Nigeria represents a significant proportion of this population. There were 18,000 new HIV infections among youth in Nigeria in 2021, which accounted for 24% of all new infections [4].

Suppressive antiretroviral therapy (ART) supports survival and increased quality of life for people living with HIV, and this requires adequate adherence to achieve viral suppression and prevent drug resistance [4]. However, medication adherence can be a challenge due to pill burden, side effects, drug fatigue, and stigmatization, among other factors, resulting in non-suppression, poor clinical outcomes, and a shortening of life expectancy [5]. Adolescents living with HIV have been identified as having particularly poor adherence to ART and poorer HIV treatment outcomes when compared to their adult counterparts [6]. In a meta-analysis involving over 50 eligible articles from 53 countries [7], the adherence level among adolescent and young adult populations was found to be poor, including among African adolescents. Studies in Nigeria have also reported varied adherence levels among adolescents and youth in different regions, with rates less than 30% in southwest Nigeria [8,9].

Mobile phone messaging may be an important strategy for improving ART adherence. In a randomized control trial among non-adherent clients (defined as adherence below 95% to ART) in a tertiary hospital in South-South Nigeria, participants received SMS-based intervention (monthly counseling and a twice-weekly mobile phone short-message service (SMS) text for four months). A majority (76.9%) of the intervention group achieved a 95% adherence level, compared to 55.8% of the control group [10]. In Nigeria, phone ownership varies with age, sex, and educational level [11]. The Nigerian Communication Commission reported more than 219 million mobile subscribers as of March of 2024 [12]. In 2016, the US Centers for Disease Control endorsed an innovative two-way text message reminder intervention (Treatment Text, TXTXT) for promoting anti-retroviral drug adherence in YLH [13]. A US study showed that 16–29-year-olds who received the TXTXT intervention were more than twice as likely to report >90% adherence to ART [14].

The TXTXT intervention plus peer navigation (iCARE Nigeria) was piloted among 40 YLH in South-West Nigeria, resulting in positive effects on viral suppression, ART adherence, and retention in care [15]. iCARE Nigeria is currently being scaled up and evaluated among 541 YLH in a stepped-wedge, randomized-controlled trial at six study sites in Nigeria [16].

This sub-study was an implementation research project conducted at one of the six iCARE study sites. This sub-study was part of iTRAIN, an adolescent HIV implementation research capacity-building program nested within iCARE Nigeria and funded by the Adolescent HIV Prevention and Treatment Implementation Science Alliance (AHISA) of the Fogarty International Center [17]. We aimed to determine the pattern of text message responsiveness as a measure of engagement with the iCARE Nigeria intervention by comparing differences across personal characteristics and across weekend/weekday and holiday/non-holiday periods. Thereafter, we explored barriers and facilitators of text message responsiveness to understand the determinants of responsiveness.

## 2. Materials and Methods

### 2.1. Description of Study Site

This study was conducted at the HIV Treatment Program at Lagos University Teaching Hospital (LUTH), which was established in 2004 with funding from the US Presidents’ Emergency Plan for AIDS Relief. The clinic has a dedicated laboratory that offers viral load and CD4+ cell count as well as hematology and chemistry assays, performed at baseline and every six months thereafter. Most patients receive their HIV prescriptions for three months, while those with unsuppressed viral loads are switched to receiving monthly prescriptions. LUTH is one of six iCARE Nigeria study sites, with two additional LUTH satellites at General Hospital Mushin and General Hospital Isolo, respectively. The iCARE Nigeria study at LUTH and its satellite sites started enrolment of participants (YLH) on 15 April 2021 and ended enrolment on 22 September 2021 with a total of 102 active participants enrolled at LUTH and its satellites.

### 2.2. Study Design and Population

This study employed a mixed-methods design, where both qualitative and quantitative data were collected. Inclusion criteria for iCARE Nigeria were an HIV diagnosis, age between 15 and 24 (with emancipation or parental consent if aged 15), current patient status at the enrollment site, ART treatment for at least 3 months, understanding of English, Hausa, Pidgin English, or Yoruba, intention to remain a patient in the clinic during the study period, and willingness to provide assent or consent. Utilizing the Dimagi CommCare provider (https://www.dimagi.com/commcare/ [accessed on 1 March 2021]), participants were sent automated, personalized, bidirectional, daily text message reminders to take their medication, followed by a personalized request to confirm adherence by text-back response (i.e., “yes” or “no”). This was finally followed by an automated encouraging message, e.g., “Way to go!” Phones were provided to those who did not have them. The focus of this analysis is on the text-back responsiveness as an indication of engagement with the TXTXT intervention.

### 2.3. Sample Size

For this sub-analysis of iCARE Nigeria data, no sampling or sample estimation was carried out as the purpose was primarily descriptive. All enrolled and active iCARE participants at LUTH and satellite sites (N = 102) were included in this iCARE sub-study.

### 2.4. Data Collection Method

#### 2.4.1. Quantitative

To assess the pattern of text-back responsiveness, secondary data were abstracted from the Dimagi Commcare web-based platform that stored the TXTXT responses for each participant from the baseline study visit to the 24-week study visit (October 2021–March 2022; approximately 180 days). We also collated participants’ baseline socio-demographic variables (age, birth sex, student status, education, and mode of HIV infection) and viral load from the iCARE Nigeria study database collected via Research Electronic Data Capture (REDCap) at Northwestern University.

#### 2.4.2. Qualitative

To explore the barriers and facilitators of text-back responsiveness, three focus group discussions (FGDs) were conducted with purposively sampled study participants. Each group consisted of 8–10 participants selected based on the level of text-back responsiveness: one group had participants with high-level responsiveness (defined as ≥70% of text-back responses to all messages requesting a response); the second group had average responsiveness (31–69% response rate); and the third group had low-level responsiveness (response rate ≤ 30%). A semi-structured focus group guide was used to explore facilitators and barriers to engagement with the TXTXT intervention. For example, “what encouraged/hindered your responsiveness to the daily [text message] reminders?” and “what are your reasons for this level of response?”. Follow-up probes were used based on participant responses. The FGDs were conducted between January and February 2023 in private rooms at LUTH and were facilitated by members of the research team (A.E.U., M.O., and O.I.). FGDs lasted an average of 50 min and were audio-recorded and transcribed for analysis.

### 2.5. Data Analysis

#### 2.5.1. Quantitative Data

Descriptive statistics of socio-demographic variables are presented as frequencies, percentages, and mean ± standard deviation. Text-back responsiveness was coded as total text-back responses divided by total text requests for response (a percentage from 0 to 100). We compared differences in text-back responsiveness by age group (15–17 versus 18–24 years), birth sex, student status, education, mode of infection, baseline viral suppression status, and weekend/weekday and holiday/non-holiday periods (with a separate analysis for public holidays and school holidays). Public holidays are official days off, recognized nationwide in Nigeria, while school holidays are breaks in the academic calendar observed by schools. During the 24-week period, we had eight days of public holidays and twenty-one days of school holidays, totaling twenty-nine days off. These variables were chosen primarily to explore their relationships with responsiveness; for example, a prior study suggested greater responsiveness on weekdays versus weekends [18]. The total number of daily responses from each participant was abstracted for analysis. Responses from participants who took their medication twice daily were recorded as “responsive” if they responded at least once for that day. Differences in responses were analyzed using *t*-tests comparing mean responsiveness and linear regression of indicators on responsiveness. Separate regression models were run for overall, weekend, public holiday, and school holiday responsiveness (controlling for demographic and treatment factors). The level of significance was set to *p* ≤ 0.05. Quantitative analysis was performed in IBM SPSS Statistics (Version 28).

#### 2.5.2. Qualitative Data

Audio recordings from the three FGDs were transcribed verbatim and then uploaded into Dedoose (Version 9.0.107), a web-based qualitative analysis software. A preliminary codebook was developed from the focus group guide with clear definitions for each code. The primary and secondary coders reviewed and revised the preliminary codes. Using a rapid content analysis approach, codes were applied to all transcripts by the primary coder and were reviewed by the secondary coders for consensus. The coding framework consisted of three main codes (facilitator, barrier, and improvement), focused on facilitators and barriers to intervention engagement. Subcodes were generated within each of the three main codes, and themes were noted. The primary coder classified major themes found across transcripts and presented the findings to the research team for consensus [19]. Each theme was distinct and had enough supporting data to be considered salient. Saturation of themes was determined when there was code clustering within and across focus groups and no emergence of new themes or sub codes. Illustrative quotes were then extracted from the transcripts to reflect the core message within the themes.

To establish rigor and increase trustworthiness of results, the team applied strategies following a framework to evaluate quality in qualitative research, using domains of credibility, dependability, confirmability, and transferability [20]. This is summarized in Table 1.

### 2.6. Ethical Considerations

Ethical approval for the iCARE Nigeria parent study was obtained from Northwestern University Ann and Robert H. Lurie Children’s Hospital and the Health Research and Ethics Committee at the Lagos University Teaching Hospital (LUTH). Ethical approval for collection of qualitative data for this sub-study was obtained from the Health Research and Ethics Committee at LUTH (approval number: ADM/DSCST/HREC/APP/5427). Written informed consent was obtained from study participants for participation in the FGDs. The confidentiality of participants was maintained by collecting FGD data by identification numbers and coded names or pseudonyms. Participants were given refreshments and transportation fare of NGN 3000 (USD 7) each.

## 3. Results

### 3.1. Quantitative Results

A total of 102 participants participated in the study. The majority were between 18 and 24 years old (63%), 67% were perinatally infected, 67% were currently in school, and approximately 54% were virally suppressed (<200 copies/mL) at baseline.

All the participants enrolled in this study responded to the TXTXT intervention at least once, and total text-back responsiveness during the 24-week intervention period was 26.5%. Among the 102 participants, 26 (25.5%) had low-level responsiveness, 50 (49%) had average-level responsiveness, and 26 (25.5%) had high-level responsiveness to the TXTXT intervention.

When comparing independent sample *t*-tests, we found a significant difference in text message responsiveness by age and education. Youths aged 18–24 years (M = 0.313, SD = 0.280) had significantly higher responsiveness compared to youth under the age of 18 (M = 0.185, SD = 0.199), and those with post-secondary level of education (M = 0.379, SD = 0.308) had higher responses than those with less than post-secondary education (M = 0.238, SD = 0.240). However, there were no significant differences in responsiveness by sex at birth, student status, viral load (at cutoffs of <200, <1000), or mode of infection (Table 2).

A paired sample *t*-test was performed to compare non-holiday versus holiday responses to text messages. There were no significant differences in responsiveness to messages received during non-holidays (M = 0.266, SD = 0.260) compared to holidays (M = 0.260, SD = 0.277); t (101) = 0.525, *p* = 0.601. The paired weekday versus weekend responses showed significantly higher responsiveness on weekdays (M = 0.277, SD = 0.268) versus weekends (M = 0.237, SD = 0.242); t (101) = −5.365, *p* < 0.001. (Table 3).

The relationship between participants’ responsiveness and socio-demographics, mode of infection, and viral load was assessed (Table 4).

In Table 4, regression models for different types of responsiveness (all responses, weekend responses, public holiday responses, and school holiday responses), controlling for age, sex at birth, student status, level of education, unsuppressed viral load, and mode of infection, are presented. Only the model for “all daily responses” approached significance (Adj R^2^ = 0.066, *p* = 0.051). The only indicator that was significantly related to responsiveness, controlling for all other factors, was age (in the models of all daily responses and school holiday responses). In both of these models, age was positively associated with responsiveness (β = 0.293; *p* = 0.032 and β = 0.298; *p* = 0.031, respectively). Education, which was significant in bivariate analysis, was not positively associated with responsiveness in any of the models.

### 3.2. Qualitative Results

The coding framework consisted of three main codes (facilitator, barrier, and improvement) focused on facilitators and barriers to responsiveness to text messages; the improvement code was applied when suggestions were made to improve the TXTXT intervention. Subcodes were generated within each of the three main codes.

A total of 48 excerpts were coded into the theme of “facilitator”. This code was applied to responses that discussed what made it easier to respond to the text message reminders. The code application of “facilitator” was applied most frequently to the focus group with high-level responders to text message reminders (45.2%), with code application at 31% for average-level responders and 23.8% for low-level responders. Sub-codes of “facilitator” included comfort, encouragement, and satisfaction. Facilitator-comfort was applied when participants discussed that they were comfortable receiving the text message reminder, and this supported intervention engagement (replying). For example, one participant described being comfortable as the text message was tailored and allowed discretion:

*What really encouraged me because in all text messages [I read] “Have you prayed?” So, I am very sure nobody has issue with me praying so anyone that picks up my phone will be like hmm ‘shey’you ‘dey’ pray like this (Pidgin tr.: so you pray like this?)...and I save the number as “baby”, so nobody had issue.*

*(Average-level responder)*

Facilitator-encouragement was applied when participants discussed being encouraged by replying to the text message, either directly through health benefits or by the message they received from the platform:

*When I finish replying or when I have replied and I get a feedback message, it was like “Oh well done, you are fantastic!” it just gives me that sense of accomplishment that I have done something well today.*

*(High-level responder)*

A total of 55 excerpts were coded into the theme of “barrier”. This code was applied to responses that discussed what made it difficult to respond to the text message reminders or what reduced engagement with the intervention. The code application “barrier” was applied most frequently to the focus group with average-level responders (46%), with code application at 34% for low-level responders and 20% for high-level responders. Sub codes of “barrier” included problems with logistics and hindering responses. Barrier-logistics was applied when participants discussed logistics such as airtime, messages being received late, and issues with mobile phones. For example, one participant described not having airtime to reply to the received message:

*Well sometimes I don’t really have airtime, so it takes a while for me to reply back; maybe when I get an airtime that’s when I will reply back.*

*(Low-level responder)*

Barrier-hinder was applied when participants discussed situations, beyond logistics, that might hinder them from replying back to the text message received, for example, being busy at work or school or being around friends when the message is received. This participant described being at work when the text messages were received:

*Most of us used to be at work and most times I am always in the kitchen so I can’t have the time to reply the messages. Maybe later, once in a while.*

*(Low-level responder)*

Another participant described not replying to the text message reminder because they were with a friend at the time:

*Sometimes I will not reply. Sometimes …, my friend will always be my side. He will be asking me what is this message for? So, I’ll not reply.*

*(Low-level responder)*

Overall, participants reported being very satisfied with the intervention. The high-level responder group discussed the importance of the intervention in supporting medication adherence and viral suppression. One participant related receiving the text messages as affirmation that others cared for them:

*It was very helpful, because I’m this kind of person that I like attention… so anytime I see the message I will be like yes, I still have people that care. I have family members that remind me though, but then just seeing the text alone, I will smile and be like okay yes, I still have people outside family that still care. So it’s very, very helpful like very, very, very helpful.*

*(High-level responder)*

Another participant requested that they continue with the intervention as they missed receiving the text message reminders:

*It was very, very satisfying. If I could even request, we should keep sending the message again because I just miss seeing the message on my phone.*

*(High-level responder)*

There were a few suggestions for intervention improvement, including providing upgraded mobile phones—specifically iPhones—providing monetary incentives for replying to texts, and increasing the amount of airtime provided.

## 4. Discussion

Quantitative analysis from our study indicated that responsiveness to bidirectional text message reminders was relatively low, and that responsiveness was higher among participants aged 18 years and over than those under 18. The overall rate of responses in our study in Nigeria (26.5%) was lower than that observed using the same platform (58%) among a similar population over a similar duration in the US [14]. This could be attributed to challenges with phone use and airtime common with digital health interventions in LMICs [21], which was also reflected by participants in our focus groups.

The disparity in responsiveness among the age groups could be attributed to the fact that adolescents aged 18 years and over could have developed a ‘thick skin’ as regards questions on their use of ART and have over time learned how to answer people who try to pry into their private affairs, including receiving and responding to daily text message reminders. On the other hand, those under 18 may not yet be as assertive, especially in response to friends or schoolmates. It also suggests that older adolescents may be better managers of their time, phones, and who they allow to access their personal space. The findings in this study also agree with the findings in a US study, which showed that older adolescents are better with adherence [14], so the findings may reflect better adherence generally. In the Nigerian context, older adolescents tend to have more control over their mobile phones than their younger counterparts, who sometimes have their phones kept by parents or guardians—an anecdotal observation in the iCARE Nigeria study.

From the qualitative analysis, the sub-codes for ‘facilitator’ included comfort, as participants affirmed that they were all comfortable receiving the text messages. Uptake of digital health interventions by youth has been reported to be influenced by the comfort experienced in using them [22]. Our findings suggest that letting youth draft their own messages may have been a strong contributing factor to this level of comfort, reducing the chance of unplanned disclosure or self-stigma. Bidirectional text messages for ART adherence among youth [14], men who have sex with men [23], and people who abuse substances [24] were also found to be acceptable in the USA. In LMICs, the effectiveness of weekly, rather than daily, bidirectional text messages in improving ART adherence has been demonstrated [25]. TXTXT was also found to be highly acceptable among youth in a pilot study in Nigeria [15]. Thus, our study findings support evidence from the previous literature that personalized, daily text message reminders can be well received by youth in LMIC settings.

Despite the provision of airtime to all YLH and a basic non-android phone to those who did not have one, to facilitate test message responsiveness, several barriers to responsiveness were logistic issues related to airtime and phone issues. It was not uncommon for YLH to use up airtime designated for responses for other means and to share phones with friends or family members.

## 5. Strengths and Limitations

Our study is one of few in Africa to assess patterns, facilitators, and barriers with respect to responsiveness to text messages for ART adherence. Our innovative design evaluated responses across weekdays versus weekends and holiday versus non-holiday periods. We also conducted assessments over a 24-week period, which was long enough to allow an appropriate evaluation of response patterns among YLH.

The generalizability of our study findings is limited because the study was conducted in only one of six study sites. Peer navigation, which was a part of the combination intervention, could also have influenced perceptions of and responsiveness to text messages among YLH in this study.

## 6. Conclusions

As an indicator of engagement with the intervention, responsiveness to daily SMS requests for confirmation of medication adherence via text message among a YLH cohort in Nigeria was relatively low. However, responsiveness was better among older participants and on weekdays and was related to identified barriers and facilitators. Our findings have some implications for research and practice. First, acceptability of digital health interventions among YLH may not translate to optimum engagement without considerations for context-specific barriers and facilitators. Second, younger YLH may require more support with interventions to promote ART adherence, and future studies should explore their particular age-specific challenges and opportunities to address them, including involving parents and caregivers. We also recommend further investigations into responsiveness disparities between, and the appropriateness of, weekday versus weekend text message reminders, as this is also critical for the implementation and efficiency of this type of intervention. mHealth platforms and interventions such as text message reminders are increasingly being leveraged to improve health outcomes among youth, especially for chronic diseases such as HIV. Details of the level of engagement and patterns of use and their related determinants will be crucial for their long-term success, in both high-income and low- and middle-income countries.

## Figures and Tables

**Table 1:** Strategies to enhance credibility and trustworthiness in the study.

Rigor Domain	Purpose	Strategies
Credibility	Establishes confidence that the perspectives shared from participants are accurate and true.	The study team was embedded within the larger research project and had knowledge and familiarity both with the study population and the intervention.
Dependability	Ensures findings are able to be replicated if the focus groups occurred within the same cohort of participants, coders, and context.	The study methods and coding procedures have been reported in detail in order for others to reproduce the study findings.
Confirmability	Establishes confidence that the results are or would be confirmed by other researchers.	Verbatim quotes have been presented to illustrate themes and codes. Member checking was performed in real time during the focus groups by summarizing what was stated and asking for confirmation. This helps establish and confirm meaning.
Transferability	The degree to which findings can be generalized to other contexts	Details about study methods and the focus group guide have been provided; while the study results should not be considered generalizable, other study teams can seek to understand barriers and facilitators to engagement with interventions among youth using the methods presented.

**Table 2:** Mean text message responsiveness by participant characteristics.

Variables	Total, (N)	%	Mean	SD	t (DF)	*p*-Value
Age (years)						
15–18	38	37.3	0.185	0.199	−2.685 (96.6) ^a^	0.009
18–24	64	62.7	0.313	0.280		
Sex at Birth						
Male	52	51.0	0.248	0.275	−0.707 (100)	0.481
Female	50	49.0	0.284	0.243		
Currently a student						
No	34	33.3	0.334	0.305	1.748 (52.1) ^a^	0.086
Yes	68	66.7	0.231	0.228		
Education						
Less than post-secondary	82	80.4	0.238	0.240	−2.218 (100)	0.029
Post-secondary	20	19.6	0.379	0.308		
Viral Load (</≥200 copies/mL threshold)						
Suppressed (viral load < 200)	55	53.9	0.287	0.284	0.974 (100)	0.358
Viremic (viral load ≥ 200)	47	46.1	0.240	0.228		
Viral Load (</≥1000 copies/mL threshold)						
Viral load < 1000	64	62.7	0.289	0.277	1.170 (100)	0.245
Viral load ≥ 1000	38	37.3	0.227	0.224		
Mode of HIV Infection						
Other Infected	34	33.3	0.297	0.286	0.862 (100)	0.391
Perinatally Infected	68	66.7	0.250	0.245		

^a^ Equal variances not assumed.

**Table 3:** Paired sample statistics of participant responsiveness to the TXTXT intervention.

Variables	Total, (N)	Mean	SD	*p*-Value	95% Confidence Interval	
					Lower	Upper
Non-Holiday versus Holiday						
Non-Holiday	102	0.266	0.260	0.601	−0.016	0.027
Holiday (Numerator/Denominator)	102	0.260	0.277			
Weekday versus Weekend						
Weekends	102	0.237	0.242	<0.001	−0.054	−0.025
Weekdays	102	0.277	0.268			

**Table 4:** Linear regression showing the relationship between text message responsiveness and socio-demographics, mode of infection and viral load.

Variables	R^2^/β	Adj R^2^	dF (6, 95)	*p*-Value
All Daily Responses	0.121	0.066	2.183	0.051
Age	0.293			**0.032**
Sex at Birth	0.051			0.602
Student	−0.032			0.780
Education	0.056			0.628
Suppressed VL	0.073			0.469
Mode of infection (perinatal)	0.045			0.668
Weekend Responses	0.101	0.044	1.178	0.112
Age	0.248			0.071
Sex at Birth	0.031			0.752
Student	−0.034			0.767
Education	0.078			0.510
Suppressed VL	0.071			0.485
Mode of infection (perinatal)	0.040			0.706
Public Holidays	0.091	0.034	1.593	0.158
Age	0.222			0.108
Sex at Birth	0.072			0.474
Student	−0.083			0.478
Education	−0.076			0.521
Suppressed VL	0.116			0.255
Mode of infection (perinatal)	−0.023			0.826
School holidays	0.098	0.041	1.712	0.127
Age	0.298			0.031
Sex at Birth	0.025			0.802
Student	−0.029			0.804
Education	0.020			0.864
Suppressed VL	0.047			0.641
Mode of infection (perinatal)	0.091			0.387

## Data Availability

Anonymized data from this analysis are available upon request and approval from the study Principal Investigators. Requests should be made to the contact Principal Investigator for the iCARE Nigeria study, Babafemi Taiwo, b-taiwo@northwestern.edu.

## Abbreviations

The following abbreviations are used in this manuscript:

iCARE	Intensive Combination Approach to Roll Back the Epidemic in Nigerian Adolescents
YLH	Youth living with HIV
ART	Antiretroviral therapy
LUTH	Lagos University Teaching Hospital
FGD	Focus group discussion
